# Strategic modulation of the gastrointestinal microbiome to enhance pancreatic cancer immunotherapy

**DOI:** 10.1016/j.drudis.2025.104528

**Published:** 2025-11-05

**Authors:** Satveer Jagwani, Laura Musumeci, Lizbeth Flores, Gerardo G. Mackenzie, Mansoor M. Amiji

**Affiliations:** 1 Department of Pharmaceutical Sciences, School of Pharmacy and Pharmaceutical Sciences, Northeastern University, Boston, MA, USA; 2 Department of Nutrition, University of California at Davis, Davis, CA, USA; 3 Department of Chemical Engineering, College of Engineering, Northeastern University, Boston, MA, USA

**Keywords:** pancreatic cancer, immunotherapy, gastrointestinal microbiome, dietary modifications, fecal microbiota transplantation, drug delivery

## Abstract

Pancreatic cancer (PC) remains one of the most lethal malignancies, characterized by aggressive progression, late detection, and limited response to current therapies. Recent research has revealed that the gastrointestinal and intratumoral microbiomes are key modulators of immune regulation, metabolism, and epigenetic pathways, influencing tumor progression and therapeutic efficacy. This review summarizes the complex microbiome–PC interplay, emphasizing microbial modulation of inflammation, immunity, and treatment resistance. We also highlight microbiome-targeted strategies, such as probiotics, prebiotics, postbiotics, and fecal microbiota transplantation, along with advanced drug-delivery platforms – including nanoparticles, engineered bacteria, and stimuli-responsive systems – for precise microbiome modulation. Integrating microbiome science with immunotherapy, nanotechnology, and epigenetic reprogramming offers promising opportunities to improve outcomes in PC.

## Introduction

Pancreatic cancer (PC) is one of the most aggressive and lethal forms of cancer, characterized by late diagnosis, rapid progression, and limited treatment options. The most common type, pancreatic ductal adenocarcinoma (PDAC), often remains asymptomatic until it reaches an advanced stage, contributing to its poor prognosis.^(p1)^ Despite advances in medical imaging and biomarker research, early detection remains a significant challenge. As a result, the five-year survival rate for PC remains around 13%, making it the third leading cause of cancer-related deaths in many countries.

In the clinic, PDAC presents several challenges. Its anatomical location deep within the abdominal cavity makes early tumors difficult to detect and surgically access. Moreover, the tumor microenvironment (TME) is highly fibrotic and immunosuppressive, which not only promotes tumor growth and metastasis, but also limits the effectiveness of chemotherapy and immunotherapy. Resistance to conventional treatments, coupled with a lack of reliable early detection methods, underscores the urgent need for novel therapeutic strategies, including those targeting inflammation, metabolic dysregulation, and the gut–pancreas axis. Understanding the complex interplay among diet, immune response, gut microbiome, inflammation, and tumor biology is increasingly being recognized as a promising avenue for improving prevention and treatment outcomes for PDAC.

Immunotherapy has emerged as a transformative approach in oncology, offering unprecedented clinical benefits in several malignancies. However, PDAC remains a notable exception, with immune checkpoint inhibitors (ICIs) such as anti-programmed death 1 (PD1), anti-programmed cell death ligand 1(PD-L1), and anti-cytotoxic T lymphocyte-associated antigen 4 (CTLA4) therapies showing minimal clinical efficacy.^(p2)^ This lack of response underscores the formidable barriers that characterize the pancreatic TME and its resistance to immune modulation.

Several intrinsic and extrinsic factors contribute to the failure of immunotherapy in PDAC. The TME is profoundly immunosuppressive, dominated by dense fibrotic stroma, regulatory T (Treg) cells, myeloid-derived suppressor cells, and tumor-associated macrophages (TAMs) that collectively inhibit cytotoxic T cell infiltration and function.^(p3)^ Additionally, PDAC tumors typically exhibit a low tumor mutational burden and poor neoantigen presentation, limiting the immune system’s ability to recognize and target malignant cells. Compounding these challenges is the physical exclusion of immune cells by the desmoplastic stroma, which acts as a barrier to both immune surveillance and therapeutic delivery. These multifaceted obstacles highlight the current research strategies that can reprogram the TME, enhance antigen presentation, and overcome immune resistance to unlock the full potential of immunotherapy in PC. One such strategy being actively investigated is the modulation of the gut microbiome.^(p4)^ Indeed, harnessing the gut microbiome to enhance immunotherapy is a highly promising strategy. This review aims to compile recent relevant information to highlight the role of the gut microbiome in PC and its intersection with immune cells. Finally, we discuss innovative drug delivery technologies to modulate the gut microbiome to harness the immune system in PC.

## Interplay between the gut microbiome and pancreatic cancer

The gastrointestinal (GI) microbiome is a complex and dynamic group of approximately 100 trillion microorganisms, composed predominantly of bacteria, but also including viruses, fungi, protozoa, archaea, and their genomic elements, which live in the digestive tract of all mammals, playing a fundamental role in the overall health of the hosts.^(p5)^

In humans, this diverse community of microbes is dominated by several major bacterial phyla, including Firmicutes, Bacteroidetes, Actinobacteria, and Proteobacteria, the relative abundances of which vary widely between individuals and are influenced by diverse factors such as diet, age, genetics, and environmental exposures.^(p6)^ These bacteria have various fundamental functions that affect host health, such as protection against pathogens, fermentation of food, immune response modulation, synthesis of essential vitamins, and metabolizing xenobiotics.^(p7),(p8)^ Over the past few years, research has increasingly focused on understanding the role of the microbiome in maintaining physiological homeostasis in humans. Disruption of microbial balance, known as dysbiosis, has been linked to the onset and progression of diseases, including cancer, as well as to drug resistance.^(p9)^

The role of the GI microbiome in cancer is complex; indeed, recent studies have indicated that gut microorganisms and their metabolites can exert both tumorigenic and antitumoral effects,^(p10)^ in addition to modulating immunotherapy efficacy.^(p11)^

Although the gut microbiome has long been implicated in PDAC development,^(p12)^ new findings have highlighted the crucial role of the microbial communities residing within the tumor itself, known as intratumoral microbiota.^(p13)^ Through migration from the gut, these microbial communities can settle into pancreatic tissue and significantly affect the TME.^(p14)^ Riquelme and collaborators^(p15)^ observed that the composition of microbes inside pancreatic tumors can influence patient survival. Indeed, individuals with higher diversity in their tumor microbiome correlated with long-term survival, together with improved outcomes. Moreover, mice that received a fecal microbiota transplant from long-term survivors showed an increase in tumor-infiltrating CD8^+^ T cells and a decrease in tumor growth compared with mice that received a fecal microbiota transplant from short-term survivors. These results suggest that microbiome profiling might have potential as a diagnostic tool and a predictive biomarker in PC management.^(p16),(p17)^

Mechanistically, specific microbial communities can influence PC outcomes by sustaining chronic inflammation and promoting immune suppression. Pushalkar *et al.*^(p18)^ provided compelling evidence that the pancreatic tumor microbiome facilitates oncogenesis by promoting both innate and adaptive immune suppression.

Not only in the case of pancreatic tumor microbiome, but also in other parts of the GI tract, the presence of certain microorganisms in a specific site can increase the chance of cancer development. This is the case, for instance, with *Porphyromonas gingivalis*, an oral bacterium that has been associated with increased PDAC risk. Indeed, Chen and collaborators^(p19)^ showed that oral inoculation of *P. gingivalis* in KC mice (LSL-K-ras^G12D^; Pdx-1-CRE transgenic mice) significantly accelerated the development of pancreatic intraepithelial neoplasia. Table 1 summarizes the principal differences between GI microbiota in healthy versus PC patients.

Beyond the microorganisms themselves, their metabolites also have crucial roles in shaping the TME and influencing therapeutic outcomes. Hezaveh *et al.*^(p20)^ demonstrated that microbial metabolism of dietary tryptophan by *Lactobacillus* produced indoles, which activated the aryl hydrocarbon receptor (AhR) in macrophages and promoted immune suppression, potentially fostering a tumor-permissive environment. Another important mechanism by which the microbiome affects PC involves the regulation of epigenetic processes, including DNA methylation, histone alterations, and non-coding RNA regulation. Microbial metabolites such as short-chain fatty acids (SCFAs), secondary bile acids, and vitamins can regulate DNA methylation and histone acetylation, thereby affecting oncogene expression and immune cell differentiation. For instance, butyrate acts as a natural histone deacetylase (HDAC) inhibitor, enhancing histone acetylation and promoting transcription of tumor-suppressive genes. Conversely, dysbiosis-driven reduction in SCFA-producing bacteria can favor hypermethylation and silencing of immune-regulatory genes. In PDAC, altered expression of key epigenetic regulators such as euchromatic histone-lysine N-methyltransferase (EHMT2), protein arginine methyltransferase 1 (PRMT1), and tumor necrosis factor-α-induced protein 1 (TNFAIP1), together with microbial shifts, has been linked to immune evasion and mammalian target of rapamycin (mTOR)–ribosomal S6 kinase (S6K) pathway activation. Collectively, these findings highlight an emerging microbiome–epigenome axis in PC progression and therapy resistance. Additionally, the microbiome contributes to chemoresistance through metabolic inactivation of anticancer agents or protection of tumor cells from therapy-induced stress. For instance, *Klebsiella pneumoniae* produces cytidine deaminase, an enzyme that inactivates gemcitabine, thereby diminishing its therapeutic efficacy.^(p21)^ Similarly, Kesh *et al.*^(p22)^ showed that in high fat diet-fed obese pancreatic tumor bearing mice, the gut microbiome was enriched with queuosine-producing bacteria that protected tumors from chemotherapy-induced oxidative stress.

Conversely, PC therapies significantly alter the gut microbiome, leading to dysbiosis, which might exacerbate treatment-related side effects. Chemotherapeutic regimens, such as gemcitabine-based ones, have been associated with reduced microbial diversity, a decline in beneficial anaerobic bacteria, and an overgrowth of potentially pathogenic species.^(p23)^ These microbial shifts can impair intestinal barrier integrity and promote systemic inflammation, collectively worsening treatment toxicity.^(p24)^ Radiation therapy further compounds these effects by directly damaging the intestinal epithelium and indirectly altering the local microenvironment, contributing to GI complications.^(p25)^

Similarly, immunotherapy with ICIs can trigger immune-related adverse events, with the GI tract being frequently affected through colitis, diarrhea, or other inflammatory conditions.^(p26)^ Altogether, these findings highlight the deeply intertwined, bidirectional relationship between microbiota and cancer therapy, emphasizing the crucial need for developing microbiome-informed strategies to mitigate treatment resistance, reduce toxicity, and ultimately improve clinical outcomes in patients with PC.^(p27),(p28)^

## Strategies for modulating the microbiome to enhance therapeutic outcomes

Given its profound influence on immune regulation and systemic physiology, the microbiome has emerged as a promising target for enhancing cancer therapy outcomes. There is growing evidence that targeted modulation of the gut microbiota can enhance therapeutic efficacy. Strategies such as dietary interventions, probiotic supplementation, and fecal microbiota transplantation (FMT) are under investigation in cancer and other diseases, such as inflammatory bowel disease and metabolic diseases.^(p29),(p30),(p31)^ Although FMT is currently FDA-approved only for recurrent *Clostridioides difficile* infection,^(p32),(p33),(p34)^ early-stage studies suggest it might have potential in enhancing immunotherapy responses in oncology, particularly in preclinical models and selected clinical trials. Nevertheless, inconsistency in study designs and microbiota profiling approaches continues to challenge broader application of microbiota-targeted strategies in cancer treatment.^(p35),(p36)^ Below, we examine emerging microbiome-targeting therapeutic strategies that have been shown to improve immune therapeutic efficacy, including dietary interventions, probiotics, prebiotics, postbiotics, FMT, and bacterial extracellular vesicles.

## Prebiotics and probiotics

Prebiotics are defined as substrates that are selectively utilized by host microorganisms, conferring a health benefit.^(p37)^ They are typically non-digestible carbohydrates (NDCs), such as inulin, fructo-oligosaccharides (FOSs), galacto-oligosaccharides (GOSs), resistant starches, and natural polyphenols, which resist digestion in the upper GI tract and reach the colon for microbial fermentation. This fermentation results in the generation of SCFAs, such as propionate, acetate, and butyrate, which promote gut health, modulate immune responses, and contribute to systemic metabolic benefits.^(p12),(p37),(p38)^

SCFAs act through multiple mechanisms: they serve as energy sources for colonocytes via transporters such as monocarboxylate transporter 1 (MCT1) and sodium-coupled monocarboxylate transporter 1 (SMCT1),^(p39)^ and they regulate immune and epithelial functions by inhibiting HDACs and activating G-protein-coupled receptors (GPR41, GPR43, and GPR109A). These epigenetic and receptor-mediated effects influence T cell differentiation, support anti-inflammatory pathways, and can reshape the tumor immune microenvironment. SCFAs have also been shown to promote interleukin-10 (IL-10) and interferon-γ (IFNγ) production under selective conditions, enhance CD8^+^ T cell cytotoxicity via inhibitor of DNA binding 2 (ID2)-dependent pathways, and suppress tumor-promoting cytokines such as IL-17 and IL-22.^(p31),(p40)^

**FIGURE 1:**
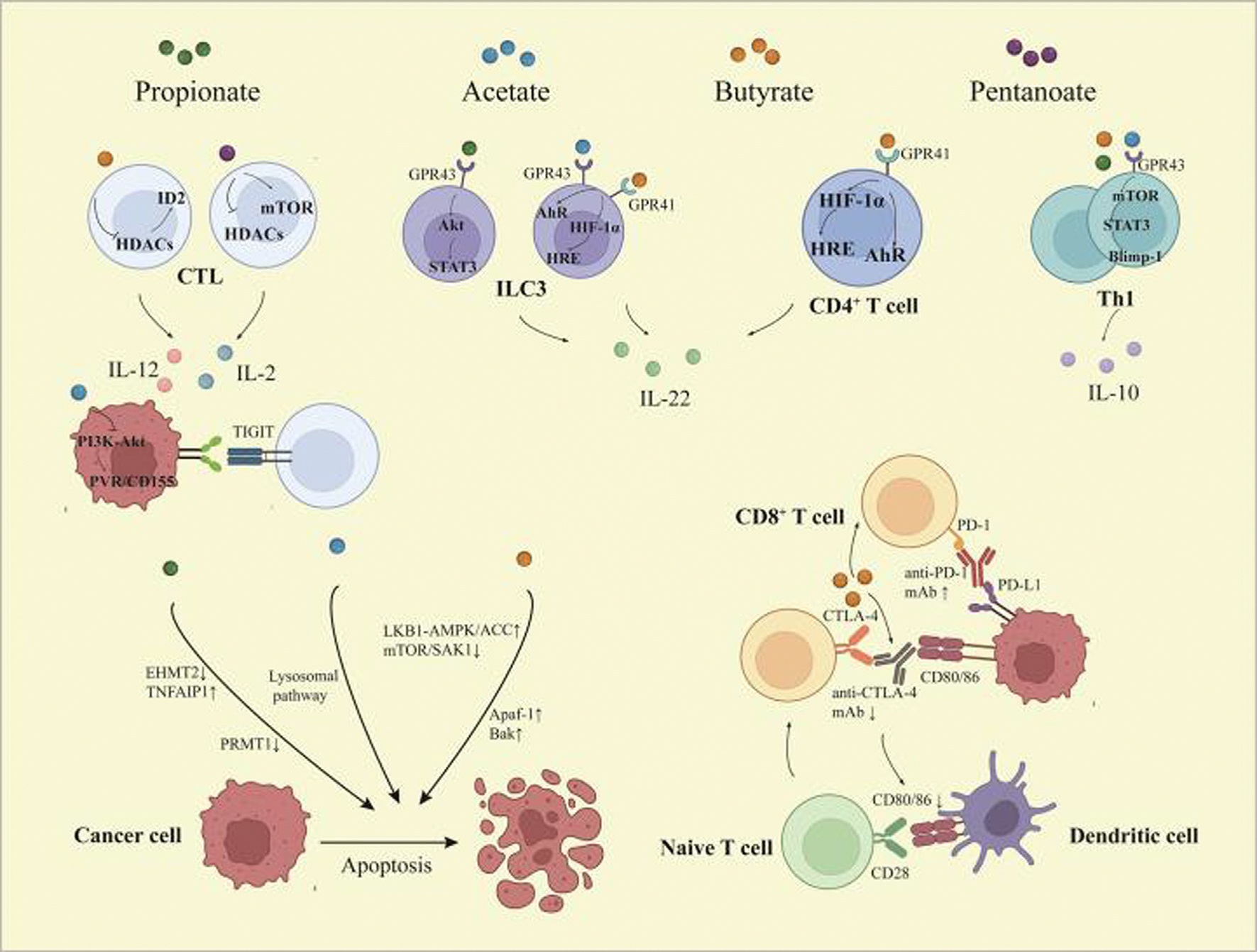
Immunomodulatory effects of short-chain fatty acids (SCFAs) within the tumor microenvironment (TME). SCFAs enhance interleukin-10 (IL-10) production in T helper 1 (Th1) cells through signal transducer and activator of transcription 3 (STAT3)- and mammalian target of rapamycin (mTOR)-mediated upregulation of B lymphocyte-induced maturation protein 1 (BLIMP1). In CD8^+^ T cells, butyrate and pentanoate promote IL-12 and IL-2 expression, respectively, via histone deacetylase (HDAC) inhibition. Acetate enhances CD8^+^ T cell responses by downregulating poliovirus receptor (PVR)/CD155 expression in cancer cells through phosphoinositide 3-kinase (PI3K)–protein kinase B (AKT) pathway suppression. SCFAs also induce IL-22 expression in CD4^+^ T cells and innate lymphoid cells (ILCs) through hypoxia inducible factor 1α (HIF1α) and aryl hydrocarbon receptor (AhR) activation or via the STAT3–mTOR axis. Although butyrate enhances anti-programmed death 1 (PD1) antibody efficacy by modulating T cell infiltration, it might impair cytotoxic T lymphocyte-associated antigen 4 (CTLA4) blockade responses and hinder dendritic cell maturation. Additionally, SCFAs contribute to tumor cell apoptosis through multiple mechanisms. Reproduced, from Ref. (p^40^).

Recent studies have also highlighted the immunomodulatory potential of SCFAs in cancer. These metabolites influence CD4^+^ T cell polarization, supporting differentiation into Treg, T helper 1 (Th1), or Th17 subsets, depending on the cytokine milieu.^(p40)^ Butyrate and propionate can induce anti-inflammatory cytokines such as IL-10 and IFNγ under specific conditions (Figure 1),^(p41)^ upregulating B lymphocyte-induced maturation protein 1 (BLIMP1) and GPR43.^(p42)^ Notably, high-fat diets suppress IL-10 and transforming growth factor-β (TGF-β) while favoring pro-inflammatory Th1/Th17 responses – a skew that SCFAs can help reverse.^(p43)^ Butyrate also enhances IL-22 expression in CD4^+^ T cells and innate lymphoid cells via hypoxia inducible factor-1α (HIF1α) and AhR activation, influencing the tumor immune microenvironment through epigenetic regulation.^(p44)^

Additionally, SCFAs modulate transcription factors, including T-box family transcription factor (T-bet), retinoid-related orphan receptor-γt (RORγT), and Forkhead box P3 (FOXP3), with low concentrations of butyrate favoring Treg development and higher concentrations promoting IFNγ production or immunosuppression.^(p45),(p46)^ In cytotoxic immunity, SCFAs such as butyrate enhance CD8^+^ T cell function by increasing expression of ID2 via HDAC inhibition, amplifying IL-12-mediated antitumor responses.^(p47)^ Pentanoate and acetate also promote effector cytokines such as tumor necrosis factor-α (TNFα), IL-2, and IFNγ,^(p48)^ while suppressing immune evasion mechanisms, such as poliovirus receptor (PVR)/CD155 via phosphoinositide 3-kinase (PI3K)/protein kinase B (AKT) inhibition.^(p49)^ Propionate has been shown to reduce IL-17 and IL-22 levels in γδT cells, dampening pro-tumor inflammation.^(p50)^

Prebiotics influence the gut microbiome in a fiber-type-specific manner, enriching distinct bacterial populations such as *Bifidobacterium* and *Lactobacillus*. The magnitude of these shifts depends on the dose and structure of the fiber.^(p51)^ Complex fibers tend to promote more specific microbial responses, often involving keystone species such as *Ruminococcus bromii*, which can facilitate cross-feeding interactions within the gut ecosystem.^(p52)^ Different fibers can direct the production of particular SCFAs, with significant physiological consequences.^(p53)^

Prebiotic compounds such as oligosaccharides (including fructans and galactans) are also being studied for their potential to prevent the adhesion of pathogenic bacteria. By mimicking host epithelial glycoconjugates, they can block pathogen adherence to intestinal cells, thereby inhibiting colonization. This mechanism is particularly relevant in cancer patients, where microbiota balance is crucial for reducing treatment-related side effects. Scientific evidence suggests that prebiotics can help to maintain a healthier microbiome during therapy, potentially enhancing treatment efficacy and minimizing toxicity.^(p54),(p55)^

Beyond modulating microbial composition and pathogen interactions, prebiotics promote the production of beneficial microbial metabolites with anti-inflammatory and antitumor properties. In the context of cancer, SCFAs and other metabolites produced through prebiotic fermentation support gut barrier function and systemic immunity.^(p38)^ Recent research is also exploring engineered prebiotic formulations designed to prolong gut retention, target specific microbial pathways, and improve therapeutic outcomes.^(p56)^

However, responses to prebiotics are highly individualized, and not all interventions yield consistent results. Some high-dose soluble fibers (e.g., inulin, pectin) have been associated with adverse effects, such as hepatic inflammation and colitis in rodent models.^(p57)^ This underscores the need for personalized approaches and careful dosing in both general health and oncology settings.

Furthermore, plant-based diets rich in whole grains, legumes, fruits, and vegetables provide naturally occurring prebiotic substrates that promote microbial diversity and SCFA production. Although the prebiotic-like effects of whole foods are promising, human studies have shown variable impacts on microbiota composition and clinical outcomes, highlighting the need for further, well-controlled trials.^(p58),(p59),(p60)^

As of now, there are no direct published reports demonstrating the efficacy of prebiotics alone in the prevention or treatment of PDAC. For instance, Abdul Rahman *et al.*^(p38)^ proposed that prebiotics can influence host metabolism and immune responses via direct modulation of microbial-derived metabolites and gut barrier function, which could potentially affect PDAC progression. Nonetheless, more targeted studies are needed to clarify these effects specifically in the context of PDAC.

In cancer settings, including PC, prebiotics might offer valuable supportive benefits by shaping a favorable gut microbiome, enhancing antitumor immunity, and potentially reducing the bioavailability of dietary carcinogens. Advances in microbiome research and prebiotic engineering continue to expand the potential for incorporating prebiotics into personalized dietary and therapeutic strategies for cancer care.

Probiotics are live microorganisms that, when administered in adequate amounts, confer health benefits to the host by enhancing gut microbial diversity and metabolic functions.^(p61)^ Traditionally, probiotics have consisted mainly of strains from *Lactobacillus* and *Bifidobacterium* genera, delivered through foods or supplements.^(p62)^ Well-studied examples, such as *Lacticaseibacillus rhamnosus* GG (LGG), have demonstrated therapeutic potential, including the treatment of *C. difficile* colitis and modulation of metabolic responses in diet-induced obesity models.^(p63),(p64)^ Recent advancements have expanded the repertoire of probiotics to include next-generation probiotics (NGPs) such as *Akkermansia muciniphila* and *Faecalibacterium prausnitzii*.^(p65),(p66)^ These novel strains, selected through genomic and functional screening, target specific disease mechanisms, but often face challenges related to viability, engraftment, and regulatory hurdles as live biotherapeutic products (LBPs).^(p37)^ Despite promising metabolic benefits observed in preclinical and early human studies, consistent, long-term alterations of the gut microbiota through probiotics remain difficult to achieve.

Several strains have demonstrated direct antitumor or immunomodulatory effects in preclinical models. For instance, *Lactobacillus casei* Shirota has been shown to stimulate natural killer (NK) cell activity and enhance expression of CD8^+^, supporting anticancer immunity.^(p67)^ *Bifidobacterium breve* has been reported to modulate dendritic cell maturation and increase tumor-infiltrating CD8^+^ T cells in murine models.^(p68)^ Moreover, *A. muciniphila* supplementation has been associated with improved responses to ICIs by enhancing antigen presentation and T cell recruitment.^(p69)^ These effects are thought to be mediated in part by microbial metabolites such as SCFAs and secondary bile acids that shape the tumor immune microenvironment.

Multi-strain consortia have emerged as an alternative strategy to enhance probiotic efficacy, leveraging microbial interactions such as lactate cross-feeding to boost beneficial metabolite production, notably butyrate. However, manufacturing and maintaining the stability of these complex formulations pose technological and regulatory challenges.^(p70),(p71)^

In the context of cancer prevention, particularly PC, probiotics might exert protective effects by binding and neutralizing dietary mutagens such as heterocyclic aromatic amines formed during the high-temperature cooking of meat.^(p72)^ Specific strains of *Lactobacillus* and *Bifidobacterium* have demonstrated the ability to bind and degrade carcinogenic compounds, potentially reducing their bioavailability and mutagenicity.^(p72),(p73)^ Furthermore, probiotics have been shown to alleviate mycotoxin and heavy metal toxicity, which are additional risk factors implicated in carcinogenesis.^(p74)^ Although further research is needed, current findings suggest that probiotics could offer a supportive strategy for modulating gut microbial balance, reducing carcinogen exposure, and promoting GI health, ultimately contributing to lower PC risk.

## Postbiotics

Postbiotics, as defined by the International Scientific Association for Probiotics and Prebiotics (ISAPP), are non-viable microbial cells, their components, or metabolic byproducts that confer health benefits to the host.^(p37),(p75),(p76)^ Derived from microbial fermentation processes, they have emerged as a promising class of therapeutic agents for modulating host physiology and immunity.^(p38)^ These include SCFAs, exopolysaccharides, bacteriocins, enzymes, cell wall fragments, organic acids, vitamins, and other bioactive molecules.^(p77)^

Unlike probiotics, postbiotics offer several advantages such as improved safety (especially in immunocompromised individuals), greater stability, and longer shelf life, making them suitable for clinical use and incorporation into food products, particularly in resource-limited settings.^(p78)^ Biologically, postbiotics exert multiple health-promoting effects, including anti-inflammatory, antioxidant, antibacterial, anti-proliferative, and immunomodulatory effects.^(p79),(p80)^ As discussed in the prebiotics section, SCFAs are key microbial metabolites that play crucial roles in host immunity and epithelial health.^(p80),(p81)^ Here, we further highlight their function in the broader context of postbiotics, which include a diverse array of bioactive microbial products.

Growing evidence suggests that postbiotics can serve as crucial mediators between the gut microbiota and host systemic immunity, influencing disease outcomes and supporting therapeutic interventions, including in PC.^(p82)^ Notably, they can act as adjuvants to reduce complications of chemotherapy and immunotherapy, such as inflammation and mucosal damage.^(p83)^

The diversity of postbiotic compounds has led to the emergence of multiple terminologies such as *para*-probiotics, pharmabiotics, and ghost probiotics, reflecting their varied sources and biological actions. Their mechanisms of action range from direct interaction with intestinal epithelial tissues to broader systemic effects. Innovative strategies are being developed to enhance their intestinal retention, control release profiles, and optimize delivery to specific target sites.^(p75)^

Prebiotics, probiotics, and postbiotics represent a triad of complementary strategies in gut microbiota modulation, each offering unique but synergistic therapeutic benefits. Prebiotics support the growth of beneficial microbes, probiotics deliver live microorganisms, and postbiotics harness the functional outputs of microbial metabolism without the risks associated with live cells. Together, they hold tremendous promise in advancing microbiota-targeted therapies for cancer, including PC, as well as metabolic, inflammatory, and autoimmune conditions. As this field continues to evolve, integrating these approaches into clinical practice could significantly enhance patient outcomes and usher in a new era of personalized, microbiome-informed medicine.

## Fecal microbiota transplantation (FMT)

FMT, first practiced in ancient China, involves the transfer of stool-derived microbial communities from a healthy donor to a recipient, aiming to restore gut microbial balance. Originally established as a highly effective therapy for recurrent *C. difficile* infection, FMT is now being explored in various GI and extraintestinal disorders associated with gut dysbiosis, including cancer.^(p12)^

FMT can be delivered using various approaches, such as colonoscopy or enema, nasogastric tube, or orally via encapsulated freeze-dried material, offering flexibility depending on clinical need.^(p84)^ Its ability to induce community-wide changes in the recipient’s microbiome and promote long-term engraftment of beneficial taxa underpins its therapeutic potential. Importantly, studies show that the donor’s microbiome composition and compatibility with the recipient are key factors in determining FMT success, giving rise to the concept of “super donors” enriched in taxa such as *A. muciniphila* and *Ruminococcus* spp.^(p85)^

In cancer models, including PDAC, FMT has shown encouraging effects. In humanized mouse models of PDAC, FMT from PDAC long-term survivors resulted in slower tumor growth, increased infiltration of cytotoxic CD8^+^ T cells, and reversal of immunosuppressive TME, compared with FMT from short-term survivors or healthy donors. Human donor-derived bacteria were detected within both gut and tumor tissues post-FMT, suggesting gut–tumor microbial crosstalk. Notably, FMT also altered tumor-associated metabolites, such as by increasing levels of the gut-derived tryptophan metabolite indole-3-acetic acid (3-IAA), which enhanced chemotherapy response in PDAC models.^(p38),(p86)^

Recent studies have extended these findings by demonstrating that a defined 11-strain bacterial consortium from healthy human microbiota could boost CD8^+^ T cell-mediated antitumor immunity and overcome resistance to chemotherapy and immunotherapy across cancer types.^(p87),(p88)^ This mechanistic insight supports the rationale for applying FMT or rationally designed microbial consortia in PDAC management.

A Phase I clinical trial (NCT04975217) is currently underway to evaluate the safety and efficacy of FMT in patients with resectable PDAC.^(p89)^ Although safety concerns, including potential pathogen transmission, remain crucial, approaches such as autologous FMT (aFMT) or refined bacterial consortia might offer safer and more controlled alternatives.^(p90)^ There are more than 100 ongoing clinical trials investigating FMT across various diseases. Positive results from these studies have laid the groundwork for expanding FMT use in oncology. PC-focused ongoing trials are summarized in Table 2.

FMT represents a promising strategy for modulating the gut–tumor–immune axis in PDAC. Although the preclinical findings are encouraging, ongoing clinical trials will be crucial for validating its therapeutic potential and determining its optimal application in PC treatment.

## Dietary interventions

**FIGURE 2:**
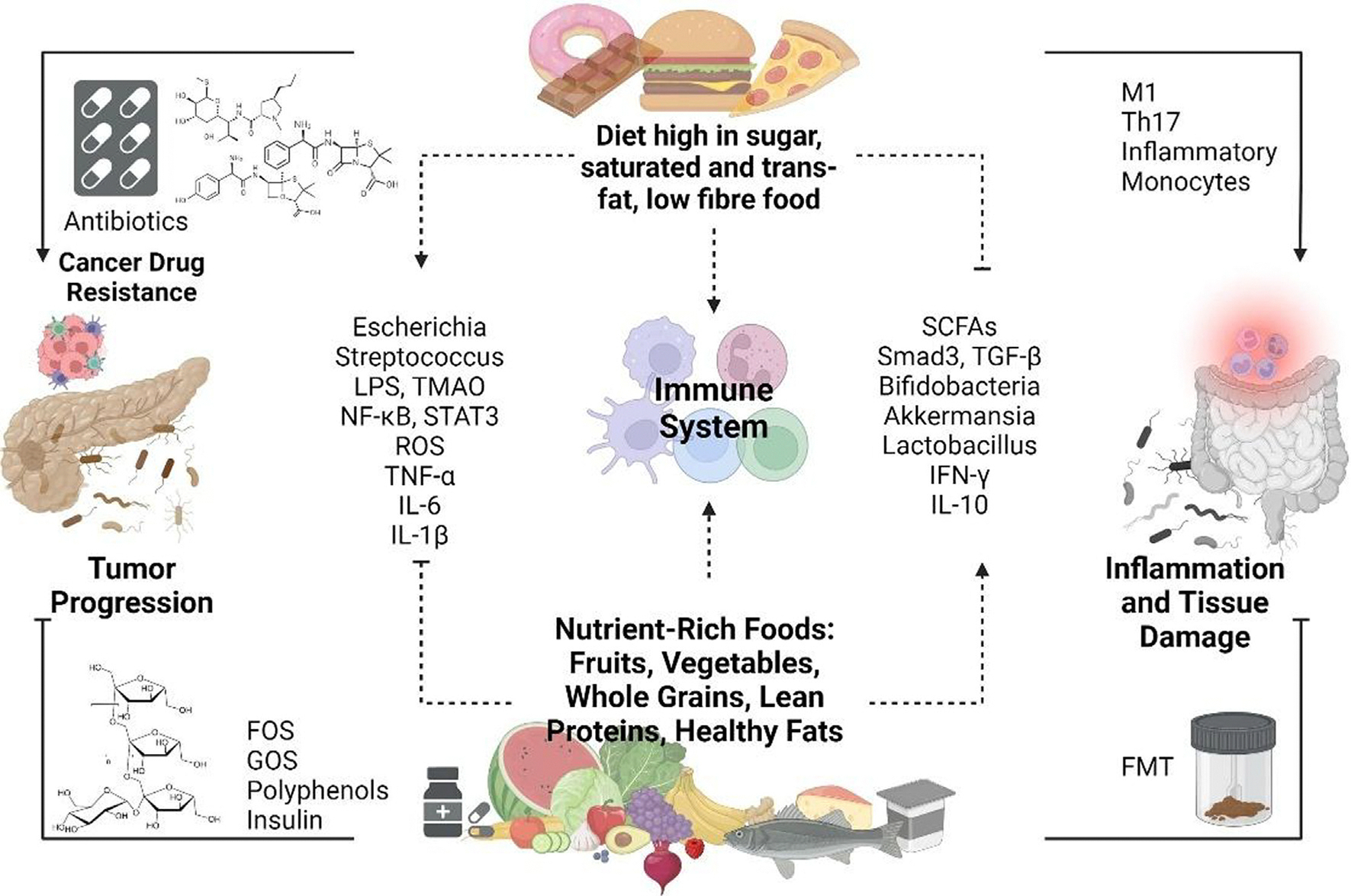
Interactions between diet, microbiota, and immune response in pancreatic cancer. This scheme highlights how dietary habits and microbial modulation influence pancreatic cancer progression and treatment outcomes. Diets high in sugar, saturated fats, and trans fats with low fiber content contribute to dysbiosis, triggering immune responses involving pro-inflammatory cytokines such as tumor necrosis factor-α (TNFα), interleukin-6 (IL-6), IL-1β, reactive oxygen species (ROS), and pathogenic bacteria (e.g., *Escherichia*, *Streptococcus*), ultimately promoting tumor progression and inflammation-mediated tissue damage. Conversely, nutrient-rich diets containing fruits, vegetables, whole grains, lean proteins, and healthy fats support beneficial microbial populations (e. g., *Bifidobacteria*, *Akkermansia*, *Lactobacillus*) and the production of useful metabolites such as SCFAs, resulting in anti-inflammatory effects, immune regulation, an increase in interferon-γ (IFNγ) and IL-10 levels, and the potential enhancement of therapeutic efficacy. Additionally, fecal microbiota transplantation (FMT) and targeted prebiotic interventions, such as fructo-oligosaccharides (FOSs), galacto-oligosaccharides (GOSs), polyphenols, and inulin are depicted as strategic approaches to restore healthy microbiota balance, reduce inflammation, and potentially counter cancer drug resistance. Antibiotics, although useful, might paradoxically contribute to dysbiosis, therapy resistance, and tumor progression. Reproduced, from Ref. (p^12^).

Diet is one of the most influential and modifiable factors shaping the composition and function of the gut microbiota (Figure 2). Dietary patterns can rapidly and profoundly alter microbial diversity, abundance, and metabolic activity.^(p91)^ High-fiber diets (typically including fruits, vegetables, and whole grains) promote the growth of beneficial gut bacteria such as *Bifidobacterium* and *Lactobacillus*, which ferment these fibers to produce SCFAs, as discussed in the prebiotics section.

Dietary fibers – defined as carbohydrate polymers resistant to digestion in the small intestine – include non-starch polysaccharides, resistant starches, and nondigestible oligosaccharides.^(p92),(p93)^ Their fermentation by gut microbes depends on properties such as solubility and degree of polymerization, influencing the production of health-promoting SCFAs and cross-feeding interactions between microbial species. These interactions help to maintain microbial diversity and intestinal homeostasis.^(p94),(p95)^

Conversely, Western-style diets, defined by excessive fat and sugar intake and limited fiber content, are associated with a reduction in microbial diversity and a dysbiotic shift toward increased numbers of Firmicutes species and decreased levels of Bacteroidetes species. Such shifts have been linked to metabolic disorders, inflammation, and carcinogenesis.^(p51),(p96)^ Diets high in animal protein and saturated fats can promote the production of harmful metabolites such as trimethylamine N-oxide (TMAO), increasing the risk of cardiovascular disease.^(p97),(p98)^

Plant-based diets, abundant in fibers and polyphenols, further enrich SCFA-producing bacteria and support anti-inflammatory microbial populations, enhancing gut health and providing a protective effect against chronic diseases.^(p75)^ Among these, dietary polyphenols – secondary plant metabolites found in foods such as green tea, berries, and red wine – have garnered attention for their dual role as microbiota modulators and anticancer agents. Poorly absorbed in the small intestine, polyphenols reach the colon, where they are metabolized by gut microbes into bioactive compounds with antioxidant, anti-inflammatory, and antitumor properties.^(p56)^ For instance, the polyphenol castalagin improved CD8^+^/FOXP3^+^CD4^+^ T cell ratios and anti-PD1 response in tumor-bearing mice.^(p99)^ Polyphenols have also been used in drug delivery systems, such as Fe^3+^ epigallocatechin gallate (EGCG)-based mucoadhesive gels and temperature-sensitive hydrogels, enhancing drug retention and efficacy in colorectal cancer and inflammation models. These findings support polyphenols as both dietary and pharmaceutical tools for microbiota-centered cancer therapies.^(p56)^

Sustained dietary habits – not just short-term interventions – are essential for establishing a stable gut microbiota that can influence cancer progression and therapeutic responsiveness.^(p91)^ Although plant-based diets rich in polyphenols, fibers, and unsaturated fats promote beneficial microbial populations (e.g., *Akkermansia* and *Faecalibacterium* spp.), Western-style diets high in fructose, cholesterol, and saturated fats are linked to dysbiosis, inflammation, and impaired gut barrier function. These microbial and metabolic shifts can dampen antitumor immunity and worsen treatment outcomes. In PDAC specifically, high-fat diets have been associated with reduced IL-10 and TGF-β levels and increased Th17/Th1 polarization – patterns that hinder ICI efficacy. In contrast, microbiota shaped by fiber- and polyphenol-rich diets produce more SCFAs, which may enhance CD8^+^ T cell activity and support immunotherapy. Although some inconsistencies persist due to dietary heterogeneity and study design limitations, mechanistic data support the concept that long-term dietary modulation of the microbiome might augment immune responses and improve outcomes in PDAC treatment.^(p100)^

Adding another layer of complexity, inter-individual differences in gut microbiota significantly modulate the impact of diet. Gut dysbiosis not only influences dietary metabolite processing, but also contributes to the pathogenesis of pancreatic and other cancers. For instance, PDAC patients exhibit distinct microbial profiles compared with healthy individuals, which could interact with diet-driven metabolic changes.^(p12),(p101)^

A ketogenic diet (KD), which is high in fat, very low in carbohydrates, and moderate in protein, induces ketosis and alters host metabolism and microbiome composition. In animal models of PC, KDs have shown antitumor and anticachexia effects, potentially through metabolic reprogramming and microbiome shifts.^(p102)^ In humans and rodents, KDs reduce overall bacterial load, but can increase *A. muciniphila* and the Bacteroidetes to Firmicutes ratio, sometimes at the cost of diversity.^(p103)^ In PDAC mouse models, feeding a KD combined with gemcitabine altered the GI microbiome, increased *Faecalibaculum* spp. and reduced *Lactobacillus* spp., which might partly explain its therapeutic benefits in PDAC models.^(p104)^ Additionally, combining KD with drugs such as eFT508 has demonstrated synergistic antitumor effects in PC models.^(p105)^ Furthermore, Ferrere *et al.*^(p106)^ provided evidence that a KD or its main ketone body, 3-hydroxybutyrate (3HB), administered intermittently, induced a T cell-dependent antitumor effect in aggressive tumor models and restored responses to ICI blockage therapies. This suggests that the KD, through 3HB, can overcome resistance to ICI block-age and enhance the effectiveness of these treatments. These findings suggest KD’s potential as an adjunct treatment approach, although long-term safety, microbial perturbations, and inter-individual variability require further study.

Overall, dietary modulation remains a promising, key strategy for influencing gut microbiota composition and cancer risk, although the inconsistencies and the interplay with microbiome variability warrant careful interpretation. A deeper understanding of nutrient–microbiota–host interactions is crucial for developing personalized dietary interventions to optimize therapeutic outcomes.

## Antibiotics and microbiome editing

Antibiotics are among the most potent modulators of gut microbiota composition. Although essential in treating bacterial infections and frequently employed during cancer care, their use leads to gut dysbiosis characterized by reduced microbial diversity, depletion of beneficial commensals (such as *Bifidobacterium* and *Lactobacillus* spp.), and potential overgrowth of pathogenic species.^(p107)^ These alterations can have profound consequences for gut barrier integrity, immune modulation, and systemic homeostasis.^(p108)^

In the context of PDAC, antibiotic use presents a double-edged sword. On the one hand, co-administering antibiotics alongside chemotherapy has been shown to enhance antitumor responses in some cases, and can improve patients’ tolerance to treatment. For instance, Weniger *et al.*^(p109)^ reported that PDAC patients with *Klebsiella pneumoniae* detected in bile cultures experienced significantly prolonged survival when treated with quinolones alongside gemcitabine-based chemotherapy. Similarly, a retrospective analysis involving >100 metastatic PDAC patients revealed that macrolide antibiotics administered for more than three days correlated with improved progression-free survival (PFS) and overall survival (OS).^(p110)^

On the other hand, other studies suggest that prolonged or inappropriate antibiotic use can be detrimental. Antibiotic-induced dysbiosis might impair antitumor immunity and promote an immunosuppressive TME.^(p12)^ Hasanov *et al.*^(p111)^ reported that tetracycline administration was linked to reduced survival in patients with resected PDAC, highlighting the complexity of antibiotic effects. Furthermore, antibiotics administered around the time of immunotherapy have been linked to reduced OS in certain cancers, probably due to the disruption of microbiota-driven immunogenic reprogramming required for optimal antitumor T-cell responses.^(p111)^

Experimental models also underscore the crucial interaction among antibiotics, gut microbiota, and cancer outcomes. In mouse models of PDAC, the beneficial effects of FMT on slowing tumor growth and enhancing CD8^+^ T-cell infiltration were abrogated when mice received antibiotics post-FMT, illustrating the microbiota’s essential role in shaping therapeutic responses.^(p112)^

Beyond these immunological and ecological disruptions, antibiotic administration also poses the serious risk of promoting antimicrobial resistance. Repeated or broad-spectrum antibiotic use can enrich for antibiotic resistance genes (ARGs) within the gut microbiome, collectively referred to as the ‘gut resistome’.^(p113)^ These ARGs might be transferred to opportunistic pathogens, contributing to the emergence of multidrug-resistant infections – an especially serious concern in immunocompromised cancer patients.^(p113),(p114)^ Chemotherapy itself might compound this issue by enhancing bacterial stress responses and horizontal gene transfer. As a result, resistant infections not only complicate clinical management, but might also worsen oncologic outcomes.^(p115)^

In summary, antibiotics represent a powerful but complex tool in PDAC management. Their ability to modulate gut microbiota offers therapeutic potential, yet risks of dysbiosis and impaired immune function caution against indiscriminate use. Future clinical studies should aim to optimize antibiotic selection, timing, and duration to harness microbiota modulation while preserving host–microbiome balance and maximizing treatment efficacy.

## Advanced delivery strategies for microbiome modulation

Effective modulation of the gut microbiome requires targeted and controlled delivery of probiotics, prebiotics, postbiotics, and microbial metabolites. These strategies must overcome challenges such as harsh gastric conditions, immune clearance, and site-specific targeting within the GI tract.^(p56),(p116)^

Microencapsulation has proven effective for protecting probiotics and postbiotics against gastric acidity and enzymatic degradation.^(p117)^ Materials such as alginate, chitosan, and lipid emulsions form protective matrices, while additives such as maltodextrin and gum Arabic enhance stability.^(p118),(p119),(p120),(p121),(p122)^ Techniques including spray drying, coacervation, and extrusion are commonly employed, with enteric coatings like Eudragit used to achieve colon-targeted release.^(p123)^

Engineered bacteria, enabled by advances in synthetic biology, can be designed to detect disease-specific cues and deliver therapeutic agents *in situ*.^(p124),(p125)^ Protective coatings (e.g., polyphenol-based nano-armor, mucin-tannic acid layers, and chitosan/alginate shells) improve mucosal adhesion and immune evasion^.(p126),(p127),(p128),(p129),(p130)^ Genetic safeguards such as kill-switches and inducible capsules further enhance safety and colonization.^(p131)^

Hydrogels, especially those based on polyphenol-protein or thermo-responsive polymers, have emerged as effective mucoadhesive carriers, enabling prolonged intestinal retention and local release of encapsulated microbes or molecules.^(p125)^

**FIGURE 3:**
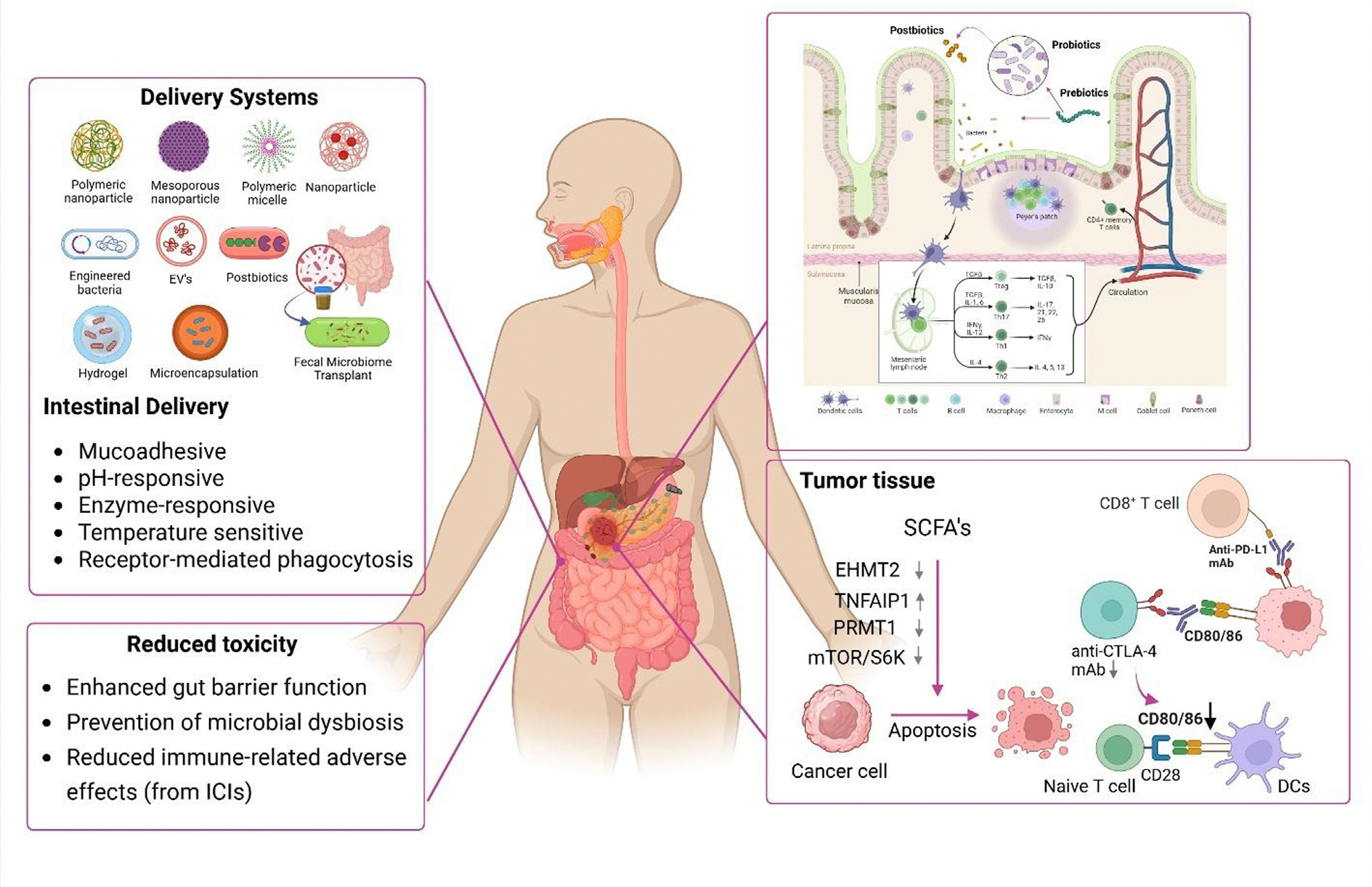
Engineered drug delivery systems targeting the gut microbiota to enhance cancer therapy and reduce toxicity. These systems – including polymeric, inorganic, lipid-based biomaterials, and extracellular vesicles – improve the intestinal delivery and bioavailability of prebiotics, probiotics, and postbiotics. By overcoming physiological barriers, they modulate the intestinal immune system through activation of antigen-presenting cells, CD4^+^ and CD8^+^ T cells, and natural killer (NK) cells, thereby restoring microbial balance, enhancing immune checkpoint inhibitor efficacy, and minimizing treatment-related toxicities. Short-chain fatty acids (SCFAs) exert additional immunomodulatory effects within the tumor microenvironment (TME) by activating signal transducer and activator of transcription 3 (STAT3) and mammalian target of rapamycin (mTOR)–ribosomal S6 kinase (S6K) pathways. They promote interleukin-10 (IL-10), IL-12, and IL-2 production in T cells, enhance cytotoxic responses via histone deacetylase (HDAC) inhibition, and downregulate immune evasion markers such as poliovirus receptor (PVR)/CD155 through phosphoinositide 3-kinase (PI3K)/protein kinase B (AKT) inhibition. These effects involve regulators such as euchromatic histone-lysine N-methyltransferase 2 (EHMT2), tumor necrosis factor-α-induced protein 1 (TNFAIP1), and protein arginine methyltransferase 1 (PRMT1), highlighting the potential of microbiome-targeted interventions in reshaping antitumor immunity. This figure was created with BioRender.com.

Nanotechnologies offer multifunctional delivery solutions using polymeric, lipidic, inorganic, or vesicle-based carriers.^(p51)^ Ligand functionalization and biomimetic coatings support targeted release, while stimuli-responsive systems triggered by tumor hypoxia, pH, redox shifts, or microbial enzymes enable spatiotemporal drug activation.^(p132)^ Bacteria–nanoparticle hybrids also exploit microbial motility for improved targeting and intratumoral drug delivery.^(p133),(p134)^ These approaches not only enhance microbial targeting and therapeutic delivery, but also modulate immune responses within the TME, as illustrated in Figure 3. In addition, engineered bacteria and bacterial extracellular vesicles (BEVs) serve as living delivery vehicles capable of colonizing hypoxic tumor regions and delivering immunomodulatory or epigenetic-modifying metabolites directly to the TME. These strategies not only enhance therapeutic bioavailability, but also allow manipulation of microbial pathways that regulate histone acetylation and DNA methylation, offering dual benefits in immune and epigenetic reprogramming. Collectively, these next-generation delivery platforms represent a promising frontier for integrating microbiome modulation with nanomedicine-based cancer therapy.

Together, these evolving delivery platforms represent a powerful toolkit for microbiome modulation in PC and beyond. A summary of key delivery strategies, materials, and applications is presented in Table 3.

## Representative examples of microbiome modulation

### Preclinical studies to improve immunotherapy response

The gut microbiome plays a crucial role in regulating host immunity and has been implicated in shaping immunotherapy outcomes. As such, modulation of the gut microbiome has emerged as a promising therapeutic strategy to improve outcomes in cancer treatment. This is particularly relevant in PDAC, where immunotherapy has shown limited efficacy. Recent preclinical studies have explored whether interventions targeting the GI microbiome could enhance immunotherapy responses in PDAC.

Several approaches have been investigated, including pharmacological interventions such as antibiotics and targeted drugs, probiotic supplementation, microbiota metabolite administration, and dietary modifications. Among these, pharmacological strategies have shown particular promise, ranging from broad-spectrum antibiotics that reshape the microbial ecosystem to targeted agents that influence specific microbial populations or metabolic pathways. For instance, Sethi *et al.*^(p135)^ demonstrated that depleting the gut microbiome using oral antibiotics reduced tumor growth in PDAC mouse models, affecting both primary tumors and liver metastases. Importantly, this effect was dependent on the adaptive immune system, because gut microbiome depletion did not reduce tumor growth in recombination activating 1 (Rag1)-knockout mice lacking mature T and B cells. Furthermore, flow cytometry analyses revealed that antibiotic treatment increased IFNγ-producing T cells, while decreasing IL-17A and IL-10-producing T cells, suggesting a shift toward antitumor immunity.

Expanding on this, Yu *et al*.^(p136)^ showed that the intestinal microbiota modulates pancreatic carcinogenesis through its effects on intratumoral NK cells. Their results highlighted how mice with a specific microbiota developed larger tumors characterized by reduced NK cell infiltration and lower IFNγ expression compared with germ-free mice. This pro-tumorigenic effect induced by the microbiota was attenuated by antibiotic treatment, but this protective effect was reversed by NK cell depletion, therefore emphasizing the crucial role of NK cells in microbiome-mediated tumor control.

Moving beyond broad-spectrum microbiome elimination, recent advances in pharmacological approaches have enabled more nuanced interventions that target specific microbial pathways or populations. For example, Zhang *et al.*^(p137)^ demonstrated that oral administration of acarbose significantly enhanced antitumor responses to anti-PD1 therapy in murine colon cancer and melanoma models. This effect was mediated through modulation of gut microbiota composition and tryptophan metabolism, ultimately promoting T cell infiltration into tumors via the upregulation of the C-X-C motif chemokine ligand 10 (CXCL10)–C-X-C motif chemokine receptor 3 (CXCR3) pathway.^(p137)^ Similarly, Luo *et al.*^(p138)^ showed that resveratrol enhanced the efficacy of anti-PD1 by increasing the relative abundance of *Desulfovibrio fairfieldensis*. Indeed, *D. fairfieldensis*, administered via oral gavage, increased the efficacy of anti-PD1 and was associated with elevated levels of prostaglandin D_2_ (PGD_2_) and infiltrating CD8^+^ T cells.^(p138)^ Deletion of collagen 1 (Col1) homotrimer in the cancer cells of KPC mice induced changes in the tumor microbiome that were associated with antitumor immune responses and sensitized PDAC to anti-PD1 therapy, leading to increased OS, an increased CD4^+^ T effector to Treg cell ratio, and CD8^+^ T cell infiltration.^(p139)^ An even more targeted approach has been developed to address intratumoral bacteria directly. Duncan *et al.*^(p140)^ developed an innovative electro-antibacterial therapy that combines electroporation with the antibiotic metronidazole to significantly enhance the clearance of intracellular bacteria, specifically *Fusobacterium nucleatum*, within PC cells.

Although pharmacological depletion and modulation of the microbiome have shown promise, an alternative approach focuses on introducing beneficial microorganisms to restore microbial balance and enhance antitumor immunity. Chen *et al.*^(p19)^ demonstrated that the oral administration of *Lactobacillus* attenuated the progression of PC promoted by *P. gingivalis* in KC mice. Probiotic treatment in these mice led to significantly reduced pancreatic weights and a marked decrease in the histological expression of epithelial–mesenchymal transition markers, such as Snail family transcriptional repressor 1 (Snail1) and zinc finger E-box binding homeobox 1 (ZEB1), as well as immunosuppressive markers including PD-L1 and galectin 3.

Building upon the success of traditional probiotic interventions, researchers have developed engineered probiotic systems designed for enhanced targeting and therapeutic efficacy. Han *et al.*^(p141)^ developed LGG probiotics functionalized with a gallium-polyphenol network, enabling targeted delivery to pancreatic tumors upon oral administration. This engineered probiotic selectively eradicated tumor-promoting Proteobacteria by disrupting bacterial iron respiration, subsequently impeding Toll-like receptor activation, reducing the expression of immunosuppressive markers, and enhancing the infiltration of cytotoxic T lymphocytes. In addition, this approach amplified the efficacy of immune checkpoint blockade in preclinical models.

Rather than introducing whole microorganisms or eliminating microbial populations entirely, another promising strategy focuses on harnessing specific metabolites produced by beneficial bacteria to enhance antitumor immunity. Indeed, numerous studies highlighted the role of specific microbial metabolites in enhancing antitumor immunity. Among these, Mirji *et al.*^(p142)^ identified TMAO, a metabolite produced by gut microbes, as a key driver of antitumor immunity in PDAC. In mice with orthotopic pancreatic tumors, TMAO administration reduced tumor growth, together with the activation of immunostimulatory TAMs and activated T cells effectors in the TME. Notably, combining TMAO with immune checkpoint blockade led to significantly improved survival compared with either treatment alone. Similarly, Luu *et al.*^(p48)^ demonstrated that the SCFAs pentanoate and butyrate enhanced the antitumor activity of cytotoxic T lymphocytes and chimeric antigen receptor (CAR) T cells by inducing metabolic and epigenetic reprogramming, specifically by enhancing mTOR activity and suppressing class I HDAC enzymes, which consequently led to increased production of effector molecules such as IFNγ and TNFα.

In another study, Hezaveh *et al*.^(p20)^ demonstrated that removing dietary tryptophan led to reduced AhR activity in TAMs, shifting them away from an immunosuppressive phenotype and promoting the intra-tumoral accumulation of TNFα^+^; IFNγ^+^; CD8^+^ T cells, indicative of an enhanced antitumor immune response. Conversely, supplementation with dietary indoles – tryptophan-derived metabolites produced by *Lactobacillus* – restored AhR activity in TAMs and reversed this effect, underscoring the crucial role of microbial tryptophan metabolism in modulating TAM function and immune responses.

Collectively, these preclinical studies underscore the intricate relationship between microbiome modulation and PC outcomes, particularly in the context of immunotherapy. They highlight a range of potential interventional strategies, from pharmacological approaches and probiotic supplementation to metabolite administration and dietary modifications. The mechanistic insights gained from these studies provide a compelling rationale for translating microbiome-targeted therapies into clinical trials, especially in combination with immunotherapy for PC patients.

### Clinical evidence and ongoing trials

Mounting clinical evidence underscores the significant role of the gut and tumor-associated microbiome in modulating treatment outcomes in PC. Several ongoing clinical trials, summarized in Table 2, are currently investigating microbiome composition, function, and modulation strategies to enhance therapeutic efficacy, mitigate treatment-related toxicity, and identify prognostic biomarkers.

Emerging studies have linked specific microbial signatures to differential responses to chemotherapy, immunotherapy, and surgical interventions. For example, altered microbial diversity and reduced abundance of commensals such as *Bifidobacterium* and *Faecalibacterium* have been associated with postoperative complications and immune dysregulation in PC patients.^(p143),(p144)^ In some trials, the gut microbiota has shown promise as a predictive biomarker for immunotherapy responsiveness and PFS.^(p145)^

Interventions under evaluation include FMT, dietary interventions, synbiotic supplementation, and targeted antimicrobial therapies.^(p89)^ Moreover, integrated multi-omic approaches combining microbiome data with transcriptomic, metabolomic, and immune profiling are being explored to refine patient stratification and develop personalized therapies.^(p15),(p146),(p147)^

## Challenges, future directions, and conclusions

Despite growing clinical evidence supporting the role of the gut and tumor-associated microbiome in PC therapy, several significant challenges hinder its full clinical translation. Inter-individual variability in microbiome composition represents a major barrier, complicating the development of standardized treatment approaches and compounded by the lack of uniform protocols for sample collection, storage, and analysis. Regulatory and ethical concerns pose additional obstacles, particularly regarding LBPs and FMT, while the complex, context-dependent nature of host–microbe–tumor interactions require sophisticated tools to understand and predict therapeutic responses. Although integrated multi-omic strategies hold promise for personalized treatment, their high cost, data integration challenges, and limited clinical availability create additional constraints, emphasizing the need for personalized approaches that account for factors such as genetics, baseline microbial composition, and prior treatments.

Future research should prioritize integrating multi-omic approaches, including metagenomics, metabolomics, transcriptomics, and immune profiling, to develop precision-based microbiome therapeutics that enable accurate patient stratification and tailored interventions. The development of synthetic microbiota consortia offers a promising alternative to conventional FMT, providing controlled and reproducible therapeutic communities with enhanced safety profiles, while advances in artificial intelligence and machine learning will enable predictive modeling of treatment responses and guide personalized therapy design. Microbiome modulation strategies, including dietary interventions, probiotics, prebiotics, postbiotics, antibiotics, FMT, and bacterial extracellular vesicles, combined with engineered drug delivery systems such as lipid-based nanoparticles and polymeric carriers, have demonstrated significant potential to restore microbial balance, support antitumor immunity, and overcome biological barriers. With continued investment in rigorous clinical trials, refined patient stratification frameworks, and interdisciplinary collaboration leveraging advances in microbiome science, immunology, and drug delivery technology, microbiome-based interventions hold transformative potential to significantly enhance treatment responses, improve patient outcomes, and usher in a new era of precision oncology for PC.

## Figures and Tables

**TABLE 1:** Comprehensive gastrointestinal microbiota differences between healthy individuals versus pancreatic cancer patients.

Category	Parameter/microbe	Healthy individuals	Pancreatic cancer patients	Clinical significance	Refs

Diversity metrics	Alpha diversity	Variable baseline	Inconsistent (higher/lower/similar)	Results vary by study methodology	(p^15^), (p^145^)
	Beta diversity	Distinct baseline structure	Significantly different from controls	Clear separation between groups	(p^145^)
	Species richness	Normal range	Increased abundance index	May indicate dysbiotic state	(p^148^)
	Duodenal diversity	Normal levels	Reduced diversity	Especially vs pancreatic cyst patients	(p^145^), (p^149^)
	*Streptococcus*	Variable	Increased risk when elevated	PDAC^a^development marker	(p^150^), (p^151^)
	*Veillonella*	Higher protective levels	Decreased	Reduced protective effect	(p^150^), (p^151^)
	*Neisseria*	Higher protective levels	Decreased	Reduced protective effect	(p^150^)
Gut microbiota - beneficial	*Bacteroides*	Higher abundance	Significantly decreased	Reduced gut barrier function	(p^152^)
	*Lachnospira*	Higher abundance	Significantly decreased	Reduced SCFA production	(p^152^)
	*Lachnospiraceae* NK4A136	Higher abundance	Significantly decreased	Loss of anti-inflammatory effects	(p^152^)
	*Bifidobacterium*	Higher abundance	Decreased	Reduced beneficial functions	(p^153^)
	Butyrate-producers	Higher abundance	Decreased (*Coprococcus, Clostridium, Blautia, Flavonifractor, Anaerostipes*)	Reduced anti-inflammatory SCFAs	(p^154^)
Gut microbiota - harmful	*Veillonella*	Lower levels	Increased abundance	Pro-inflammatory effects	(p^155^)
	*Klebsiella*	Lower levels	Increased abundance	Pro-inflammatory effects	(p^155^)
	*Selenomonas*	Lower levels	Increased abundance	Pro-inflammatory effects	(p^155^)
	LPS-producing bacteria	Lower levels	Increased (*Prevotella, Hallella, Enterobacter*)	Enhanced systemic inflammation	(p^155^)
	*Acinetobacter*	Lower levels	Significantly elevated	Positive correlation with CA242	(p^152^)
	*Delftia*	Lower levels	Increased	Correlated with tumor markers	(p^156^)
	*Fusobacterium*	Variable	Increased	Correlated with tumor markers	(p^156^)
	*Escherichia/Shigella*	Lower levels	Increased	Correlated with tumor markers	(p^157^)

a Abbreviations: LPS, lipopolysaccharide; PDAC, pancreatic ductal adenocarcinoma; SCFA, short-chain fatty acids.

**TABLE 2:** Microbiome-related clinical trials in the treatment of pancreatic cancer.

Study title (ClinicalTrials.gov identifier)	Study objective	Disease condition	Trial phase	Intervention/samples	Current Status

Fecal Microbial Transplants for the Treatment of Pancreatic Cancer (NCT04975217)	Interventional study evaluating safety, feasibility, and microbiome-related changes in the gut, oral cavity, and TME^a^, as well as associated immunological responses, following FMT in patients with resectable PDAC	PDAC	Early Phase I	FMT capsule and therapeutic colonoscopy	Recruiting
CHemotherapy and Stool Transplant in PDAC (CHASe-PDAC) (NCT06393400)	Interventional study evaluating the safety of combining oral FMT with gemcitabine and nab-paclitaxel as first-line therapy in patients with advanced PDAC, and to explore associated microbiome, immune, and clinical outcomes	Advanced PDAC	Phase I	FMT capsule, PEG3350, gemcitabine, and nab-paclitaxel/blood, stool	Recruiting
Microbial Diversity of Pancreatic Diseases (NCT03809247)	Observational study identifying microbial differences and potential biomarkers linked to pancreatic diseases by comparing gut flora across patient and healthy groups	PC and other pancreatic diseases	-	Stool, other digestive secretions, blood and tissue samples in case of surgical resections	Recruiting
Modulation of the Gut Microbiome With Pembrolizumab Following Chemotherapy in Resectable Pancreatic Cancer (NCT05462496)	Interventional study assessing the immune activation and safety of combining antibiotics and pembrolizumab with neoadjuvant chemotherapy in resectable PC	PC	Phase II	Folfirinox, ciprofloxacin, metronidazole, pembrolizumab, and surgical resection/stool and blood	Recruiting
ARGONAUT: Stool and Blood Sample Bank for Cancer Patients (NCT04638751)	Observational study evaluating the potential of microbiome composition to predict survival outcomes and immune-related biomarkers	PC, NSCLC, CRC, and TNBC	-	Blood and stool	Recruiting
Intestinal Microbiota Impact for Prognosis and Treatment Outcomes in Early Luminal Breast Cancer and Pancreatic Cancer Patients (NCT05580887)	Observational study assessing whether specific gut microbiome patterns are associated with poor or favorable treatment outcomes using 16S rRNA sequencing	Breast cancer and PC	-	mFolfirinox, doxorubicin, cyclophosphamid, paclitaxel, and carboplatin/blood, stool, and tumor tissue	Recruiting
Volatiles in Breath and Headspace Analysis - Diagnostic Markers (Volatolome) (NCT03228095)	Observational study assessing VOC-based technologies for noninvasive diagnosis and monitoring of digestive tract and infectious diseases	Including PC among various cancers and inflammatory diseases	-	Blood and biopsy material	Enrolling by invitation
Association of Plant-based Diet and Gut Microbiome Signature with Response to Neoadjuvant Therapy in Pancreatic Ductal Adenocarcinoma (NCT06595160)	Observational study assessing the relationship between pretreatment dietary patterns, fecal microbiome diversity, and chemotherapy response in patients with operable PDAC	PDAC	-	Stool	Recruiting
Oral Immunonutrition With Synbiotics, Omega 3 and Vitamin D in Patients Undergoing Duodenopancreatectomy for Tumoral Lesion (SIO3D) (NCT05271344)	Interventional study assessing whether enhanced immunonutrition with synbiotics, omega-3, and vitamin D reduces postoperative morbidity compared to standard immunonutrition in patients undergoing duodenopancreatectomy	PC	NA	Nutritional products/stool and blood	Recruiting
Effects of Peptamen 1.6 in Malnourished Patients (or At Risk) with Pancreatic Neoplasia Undergoing Cephalic Pancreaticoduodenectomy (CPD): a Mechanistic Study (NCT06852014)	Interventional study comparing Peptamen 1.6 and Resource HP/HC in malnourished PC patients undergoing pancreaticoduodenectomy, evaluating metabolic, inflammatory, and digestive outcomes through clinical and organoid-based analyses	PC and CPD	NA	Nutritional supplement/blood and stool	Recruiting
The Characteristics of Oral Microbiota in Chronic Pancreatitis and Autoimmune Pancreatitis (NCT07015580)	Observational study characterizing oral microbiota changes in chronic pancreatitis and comparing microbial profiles with those in PC and autoimmune pancreatitis using full-length 16S rRNA sequencing	PC and pancreatitis	-	Saliva	Recruiting
Clinical Study on Fecal Microbiota Transplantation for Diarrhea After Total Pancreatectomy (FMT-TP) (NCT06960122)	Interventional study assessing the safety and efficacy of oral fecal FMT in reducing post-total pancreatectomy diarrhea in PC patients, based on clinical outcomes and gut microbiota composition	PC	NA	FMT capsule/stool	Not yet recruiting
The Effect of Probiotics ATG-F4 in Cancer Patients (NCT06436976)	Interventional study evaluating the effect of Lactobacillus reuteri ATG-F4 on chemotherapy-induced side effects and cachexia in patients with CRC and PC	PC and CRC	Phase II	LT-002 (*L reuteri* ATG-F4)/stool and blood	Recruiting

a Abbreviations: CPD, cephalic pancreaticoduodenectomy; CRC, colorectal cancer; FMT, fecal microbiota transplantation; NA, not applicable; NSCLC, non-small cell lung cancer; PC, pancreatic cancer; PDAC, pancreatic ductal adenocarcinoma; PEG, polyethylene glycol; TME, tumor microenvironment; TNBC, triple negative breast cancer; VOC, volatile organic compound.

**TABLE 3:** Summary of delivery technologies for modulation of the gastrointestinal microbiome.

Delivery strategy	Material/technology	Key features	Applications	Refs

Microencapsulation	• Calcium alginate/freeze drying • Acrycoat S100/spray drying • Pectin and hi-maze inulin/freeze drying Sodium alginate and FOS^a^/extrusion • Chitosan and gelatin/emulsion • Alginate solution and CaCl_2_/ impinging aerosol technique • Eudragit S100/spray drying	Protects probiotics/postbiotics from gastric acid and enzymes; enables colon-targeted delivery; improves shelf stability	Targeted probiotic delivery; gut microbiome modulation; food functionalization	(p^117^), (p^118^), (p^158^), (p^159^), (p^160^), (p^161^)
Engineered bacteria	• EcN strain • *Listeria monocytogenes, Bacteroides* • Tannic acid and mucin coated (LbL) EcN • Chitosan and alginate coated (LbL) EcN	Synthetic biology-enabled; integrated biosensors and gene circuits; responsive to disease cues; include kill-switches, inducible cloaking, and LbL coatings	Localized drug delivery; immunotherapy enhancement (e.g., PD-L1 response); modulation of tumor vasculature; gut colonization; real-time biomarker sensing; microbiota reprogramming	(p^125^), (p^126^), (p^162^), (p^163^), (p^164^)
Nanotechnology	• Polymeric/lipid nanoparticles • Inorganic carriers • Extracellular vesicles • Dextran-based NPs, ligand/biomi-metic functionalization	Stimuli-responsive release (pH, redox, enzyme, hypoxia); hybrid bacteria-NP systems for tumor targeting	Microbiota modulation; chemotherapy co-delivery; immunotherapy enhancement	(p^56^), (p^132^), (p^133^), (p^165^), (p^166^)
Hydrogels	• Poloxamer F127-DA • PDB-alginate-Ca • Mucosal adhesives, temperature-sensitive polymers	Mucoadhesive; temperature/pH-responsive; enables localized and sustained release; biocompatible	Used for colonic drug delivery, improving retention of therapeutic agent, modulating local inflammation, and inhibiting colitis-associated cancer	(p^125^), (p^167^), (p^168^), (p^169^)

a Abbreviations: EcN, *E. coli* Nissle 1917; FOS, fructo-oligosaccharide; LbL, layer-by-layer; NP, nanoparticle; PDB, 1,4-bi(phenylalanine-diglycol)-benzene; PD-L1, programmed cell death ligand 1.

## Data Availability

No data was used for the research described in the article.
